# Development and evaluation of a centrifugal disk system for the rapid detection of multiple pathogens and their antibiotic resistance genes in urinary tract infection

**DOI:** 10.3389/fmicb.2023.1157403

**Published:** 2023-05-02

**Authors:** Nianzhen Chen, Gen Li, Yuying Si, Yangqin Ye, Tong Zhang, Dali Chi, Wenyan Zhang, Lifeng Pan, Guoying Qu, Yun Lu, Ming Zong, Guodong Sui, Lieying Fan

**Affiliations:** ^1^Department of Clinical Laboratory, Shanghai East Hospital, Tongji University School of Medicine, Shanghai, China; ^2^Shanghai Key Laboratory of Atmospheric Particle Pollution and Prevention (LAP3), Department of Environmental Science and Engineering, Fudan University, Shanghai, China; ^3^Fosun Diagnostics Co., Ltd., Shanghai, China; ^4^Department of Microbiology, Pudong New Area Center for Disease Control and Prevention, Shanghai, China; ^5^Weifang Community Health Service Center, Shanghai, China; ^6^Huamu Community Health Service Center, Shanghai, China

**Keywords:** LAMP, centrifugal disk, urinary tract infection, pathogens, antibiotic resistance genes

## Abstract

**Background:**

Urinary tract infections (UTIs) are some of the most common bacterial infections in the world. Nevertheless, as uncomplicated UTIs are treated empirically without culturing the urine, adequate knowledge of the resistance pattern of uropathogens is essential. Conventional urine culture and identification take at least 2 days. Here, we developed a platform based on LAMP and centrifugal disk system (LCD) to simultaneously detect the main pathogens and antibiotic resistant genes (ARGs) of urgent concern multidrug-resistant among UTIs.

**Methods:**

We designed specific primers to detect the target genes above and evaluated their sensitivity and specificity. We also assessed the result of our preload LCD platform on 645 urine specimens with a conventional culturing method and Sanger sequencing.

**Results:**

The results obtained with the 645 clinical samples indicated that the platform has high specificity (0.988–1) and sensitivity (0.904–1) for the studied pathogens and ARGs. Moreover, the kappa value of all pathogens was more than 0.75, revealing an excellent agreement between the LCD and culture method. Compared to phenotypic tests, the LCD platform is a practical and fast detection approach for methicillin-resistant *Staphylococcus aureus*, vancomycin-resistant *Enterococci*, carbapenem-resistant *Enterobacteriaceae*, carbapenem-resistant *Acinetobacter*, carbapenem-resistant *Pseudomonas aeruginosa* (kappa value of all >0.75), and non-extended-spectrum β-lactamase producers.

**Conclusion:**

We developed a detection platform that has high accuracy and that meets the need for rapid diagnosis, which can be completed within 1.5 h from specimen collection. It may be a powerful tool for evidence-based UTIs diagnosis, which has essential support for the rational use of antibiotics. More high-quality clinical studies are required to prove the effectiveness of our platform.

## 1. Introduction

Urinary tract infections (UTIs) are some of the most frequent community and nosocomial-acquired infectious diseases affecting up to 150 million people worldwide annually (Harding and Ronald, 1994; Stamm and Norrby, 2001); indeed, 50% of females will experience a UTI at least once during their lifetime (Davenport et al., 2017). Clinically, UTIs are usually characterized by frequent urination, urgency, urinary dysfunction, and poor urination (Foxman, 2014). However, it is likely that these symptoms will lead to upper or lower UTIs, which will develop into pyelonephritis or cystitis if left untreated (Momtaz et al., 2013; Flores-Mireles et al., 2015; Spaulding and Hultgren, 2016). Even worse, the bacteria may be spread to the blood and cause a life-threatening infection if treated inappropriately (Flores-Mireles et al., 2015). Hence, a timely and efficient diagnosis is crucial for prompt therapy.

UTIs are caused by both Gram-negative (GN) and Gram-positive (GP) bacteria, as well as by certain fungi (Flores-Mireles et al., 2015). According to national bacterial resistance monitoring data of the China Antimicrobial Surveillance Network (CHINET), the isolated bacteria in urinary tract specimens mainly included *Escherichia coli* (Eco), *Klebsiella pneumoniae* (Kpn), *Acinetobacter baumannii* (Aba), *Pseudomonas aeruginosa* (Pae), *Proteus mirabilis* (Pmi)*, Enterobacter cloacae* (Ecl), *Staphylococcus* spp., *Enterococcus* spp., and *Candida* spp. The emergence of multidrug-resistant (MDR) bacterial pathogens is highlighted as a threat to healthcare systems worldwide by the World Health Organization (WHO) (Toner et al., 2015). Recently, the Centers for Disease Control and Prevention (CDC) of the United States published the 2019 Antibiotic Resistance Threat Report, defining the MDR into four categories (CDC, 2019). They emphasize that there is no safe place for antibiotic resistance and suggested that the most frequent urgent and serious threats of MDR pathogens among UTIs are carbapenem-resistant *Enterobacteriaceae* (CRE), carbapenem-resistant *Acinetobacter* (CRAB), extended-spectrum β-lactamase (ESBL)-producing *Enterobacteriaceae*, methicillin-resistant *Staphylococcus aureus* (MRSA), and vancomycin-resistant *Enterococci* (VRE). Additionally, carbapenem-resistant Pae (CRPA) is divided into the most critical threats of MDR by the WHO.

Currently, the diagnosis of UTIs is usually based on clinical signs and symptoms, although laboratory testing is also necessary for accurate diagnosis. Urine strips or dipsticks, urinary sediment microscopy, and urine culture are currently the primary diagnostic assays for UTIs. The dipstick urinalysis methods for nitrite and/or leukocyte esterase in patient urine samples are rapid, simple, and inexpensive but insensitive (Semeniuk and Church, 1999; Wilson and Gaido, 2004). Conventional urine culture and identification are the “gold standard” to confirm the diagnosis of UTIs. However, this takes at least 2 days, which may lead to diagnostic and evidence-based treatment delays (Wilson and Gaido, 2004; Yan et al., 2011). Clinically, empiric antimicrobial therapy is initiated prior to the results of the culture and antibiotic susceptibility testing (AST). Therefore, the drug resistance phenomenon among bacterial pathogens has become increasingly severe. It is important to develop fast and accurate methods to identify different pathogens, as well as drug resistance status for accurate diagnosis and treatment.

In recent years, molecular biology techniques, including fluorescence *in situ* hybridization (FISH), matrix-assisted laser desorption/ionization time-of-flight mass spectrometry (MALDI-TOF MS), and polymerase chain reaction (PCR), have been used to detect pathogenic microorganisms, which has improved the timeliness and precision over existing clinical methods (Wu et al., 2010; Fairfax and Salimnia, 2013). Although extensive research has been conducted on molecular perspective, only a few studies have directly detected pathogenic bacteria from samples and evaluated their drug resistance without separating bacteria in advance (Goudarzi et al., 2019). Numerous manufacturers have created multiplexed *in vitro* diagnostic (IVD) platforms during the past 10 years that can identify the nucleic acid profiles of many of the infectious disease-causing pathogens (Gadsby et al., 2010; Poritz et al., 2011; Pierce and Hodinka, 2012; Butt et al., 2014; Lee et al., 2017; Ramanan et al., 2018). Here, we introduce a new platform for directly detecting pathogens and their antibiotic resistant genes (ARGs) from urine samples based on loop-mediated isothermal amplification (LAMP) and a centrifugal disk system (LCD).

In 2000, Notomi et al. established the LAMP method (Notomi et al., 2000) to rapidly amplify DNA or RNA targets under isothermal circumstances. The LAMP method constitutes a simple, rapid (within 45 min), and inexpensive detection technique with high sensitivity and specificity (Nagamine et al., 2002). Taking advantage of the LAMP reaction, it has frequently been combined with a centrifugal device for rapid detection of various pathogens (Hiltunen et al., 2018; Wang et al., 2018), which is applied to point-of-care testing (POCT) to facilitate the control of epidemics. However, few studies have simultaneously realized the rapid detection of multiple pathogens and their ARGs.

**Figure 1:**
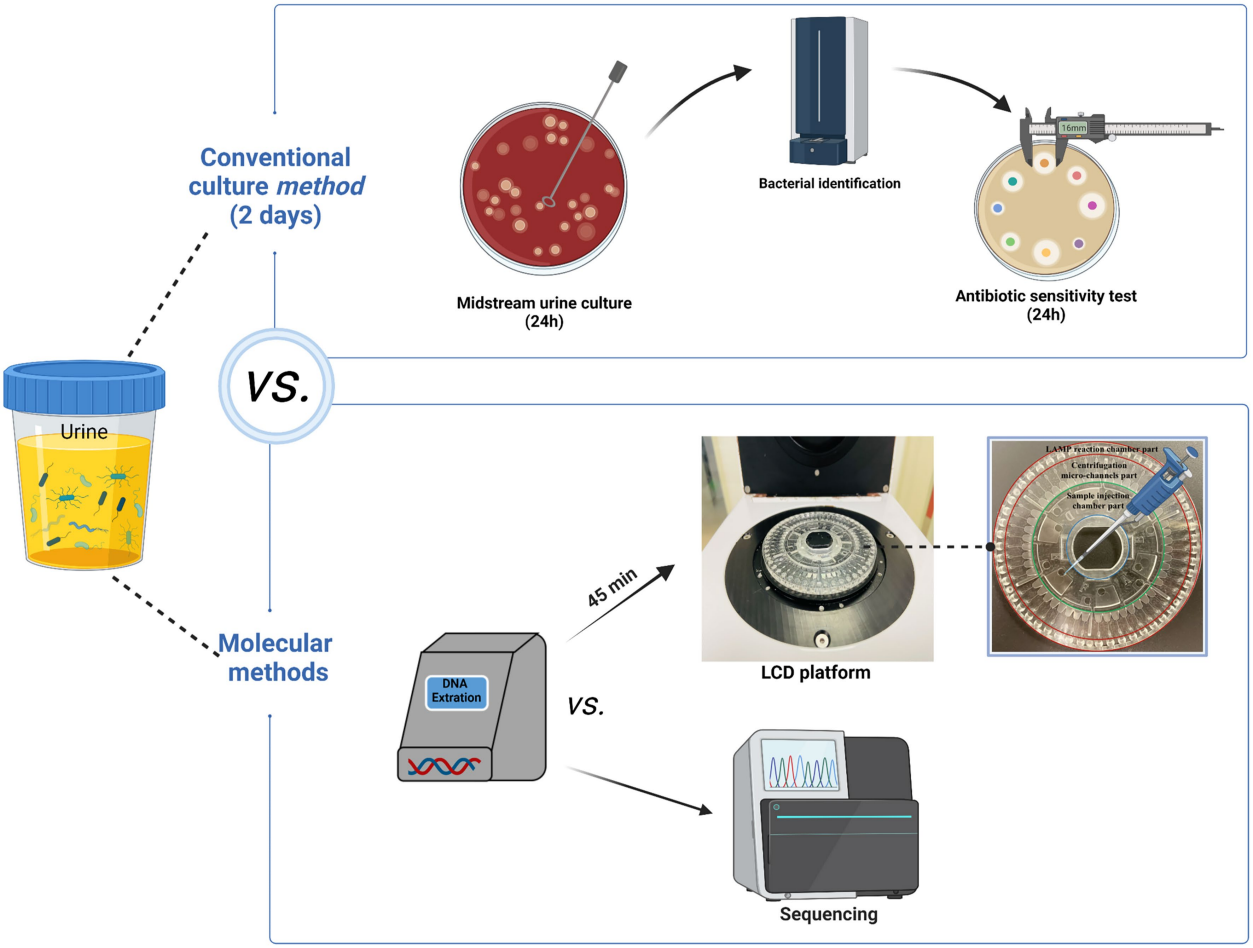
The specifics of the entire procedure. The entire detection procedure of LCD platform was finished within 1.5 h. Figure generated with bioRender.

Controlling antibiotic resistance by combating the problem of antibiotic misuse requires reliable AST results. Therefore, we developed a centrifugal disk system integrated with the LAMP method for the simultaneous rapid and accurate detection of 10 pathogens and 14 ARGs among UTIs. The targeted pathogens included Eco, Kpn, Aba, Pae, *S. aureus* (Sau), Pmi, Ecl, *Enterococcus faecalis* (Efa), *Enterococcus faecium* (Efm), and *Candida albicans*. Goals of the AGRs including SHV, TEM, CTX-M-1 group, CTX-M-9 group, KPC, NDM, IMP, OXA-23, OXA-24, OXA-48, DHA, CMY, VanA, and mecA. We also explored the performance of the LCD platform in clinic. A schematic diagram of the full procedure is shown in Figure 1.

## 2. Materials and methods

### 2.1. Clinical specimens and quality control

Patients in the menstrual period and urine samples with polymicrobial (≥3) culture were excluded, as were repeat samples from the same patients. Therefore, a total of 645 midstream urine specimens were collected from patients who showed signs and symptoms of UTIs (such as frequency, urgency, fever, difficulty urinating, and pain with urination) and were hospitalized at Shanghai East Hospital, School of Medicine, Tongji University, between September 2019 and October 2021. The protocol was approved by the “Ethics commission of the Shanghai East Hospital” under the protocol no. 2019-021. All of the samples were residual specimens after performing conventional microbiological culture and AST. All samples were kept at 4°C for a maximum of 2 h prior to further processing (DNA extracting).

The following 10 standard strains were purchased from Shanghai Beinuo Biotechnology Co., Ltd. (Shanghai, China): Eco (ATCC 35218), Kpn (ATCC 13883), Aba (ATCC 19606), Pae (ATCC 27853), Sau (ATCC 25923), Pmi (ATCC 12453), Ecl (ATCC 13074), Efa (ATCC 29212), Efm (ATCC 35667), and *C. albicans* (ATCC 90028). Additionally, 19 “standard strains” collected from the Microbiology Laboratory of Shanghai East Hospital, which were validated by whole-genome sequencing to produce different drug-resistant genotypes, were used as positive controls (Supplementary Table S1).

### 2.2. Hospital laboratory tests

All operations were performed in strict accordance with the 4th edition of the National Clinical Laboratory Procedures (Shang et al., 2015). The qualifying urine samples were inoculated on blood agar and MacConkey agar plates (Thermo Fisher Scientific, KS, United States) using a 0.001 ml inoculation loop under sterile conditions. The colonies were counted after being incubated for 24 h at 37°C and 5% CO_2_. A positive urine culture was defined as colony count ≥10^5^ colony-forming units (CFU)/mL for GN bacteria or ≥10^4^ CFU/ml for GP bacteria or *Candida* spp. Subsequently, bacterial identification was determined using Microflex^®^ LT/SH MALDI-TOF mass spectrometer and MALDI Biotyper version 2.3 (Bruker, Germany) according to the manufacturer’s instructions, which was conducted in triplicate for each strain. The VITEK^®^ 2 compact automatic microbiological analyzer (version 8.0, bioMérieux, Marcy l’Etoile, France) or the manual Kirby–Bauer (K–B) disk diffusion method was used to perform AST, and the results were assessed based on the Clinical and Laboratory Standards Institute document (CLSI, 2019). CRE, CRAB, and CRPA are defined as being resistant to imipenem or meropenem (the minimal inhibitory concentration [MIC] of imipenem or meropenem ≥4 μg/ml was defined as CRE; MIC of imipenem or meropenem ≥8 μg/ml was defined as CRAB or CRPA). ESBL producers were identified by determining the MIC for ceftazidime (CAZ-MIC ≥16 μg/ml) or cefotaxime (CTX-MIC ≥4 μg/ml). Cefoxitin susceptibility testing was used to identify MRSA (MIC ≥4 μg/ml). VRE was screened according to susceptibility to vancomycin (MIC ≥32 μg/ml).

### 2.3. Design of LAMP primers

As shown in Supplementary Tables S2, S3, 24 target LAMP primer sets for UTIs were designed with reference to the published sequences in GenBank and using the online software Primer Explorer V5 (Eiken Chemical Co., Ltd., Tokyo, Japan).^1^ These primers include an outer forward primer (F3), an outer backward primer (B3), a forward inner primer (FIP), and a backward inner primer (BIP). A loop forward primer (LF) and/or a loop backward primer (LB) were also designed to accelerate the reaction. All primer pairs were synthesized by Sangon (Sangon Biotech Co., Ltd., Shanghai, China). The primers were formulated into a solution and stored at −20°C as described in our previous study (Chen et al., 2021; Si et al., 2021), which contained 0.2 μM of outer primers (F3 and B3), 1.6 μM of inner primers (FIP and BIP), and 0.6 μM of loop primers (LF and LB).

### 2.4. Synthesis of the LCD platform

**Figure 2:**
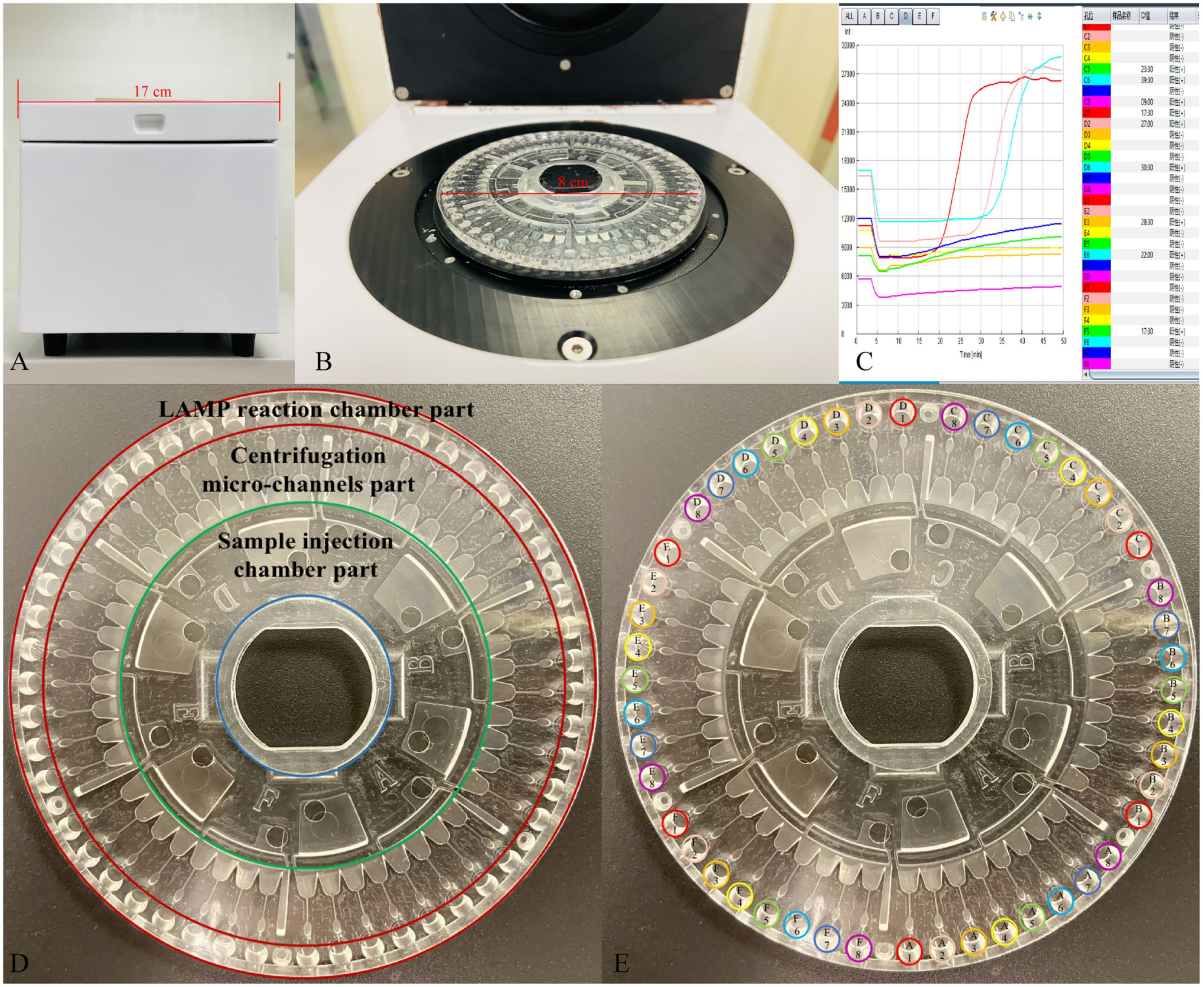
The developed centrifugal disk system, real-time detection instrument, and representative real-time amplification curves. **(A,B)** Photograph of a detection instrument. **(C)** Real-time amplification curves. **(D)** Simple composition of the centrifugal disk system. **(E)** The preload buffer and primers order of centrifugal disk. A1/D1: Eco; A2/D2: Kpn; A3/D3: Aba; A4/D4: Pae; A5/D5: Sau; A6/D6: Pae; A7/D7: Ecl; A8/D8: Efa; B1/E1: Efm; B2/E2: *C. albicans*; B3/E3: SHV; B4/E4: TEM; B5/E5: CTX-M-1 Group; B6/E6: CTX-M-9 Group; B7/E7: KPC; B8/E8: NDM; C1/F1: IMP; C2/F2: OXA-23; C3/F3: OXA-24; C4/F4: OXA-48; C5/F5: DHA; C6/F6: CMY; C7/F7: mecA; C8/F8: VanA.

The LCD platform examined in this study included a disposable 48-channel centrifugal disk and a real-time detection instrument. As shown in our previous study (Liu et al., 2018) and in Figure 2D in this study, the centrifugal disk simply consists of the following three parts: a sample injection chamber part, a centrifugation micro-channel part, and a LAMP reaction chamber part. The goal of this platform was to simultaneously detect 24 target genes in each urine sample. To this end, we employed the preset-gel method for the LAMP chamber part as follows: 2.5 μl 10× ThermoPol reaction buffer (New England Biolabs Ltd., Beijing, China), 9.5 μl mixture (with ddH_2_O and 8 mM Mg^2+^), and 1 μl SYBR-Green I (Beijing Solarbio Science & Technology Co., Ltd., Beijing, China) were mixed and then low melting point agarose (Sangon Biotech Co., Ltd., Shanghai, China) was added at a mass volume ratio of 5/1000 (g/mL). Subsequently, the primer solution of different targets was added in the order shown in Figure 2E when the above system was heated to 60–70°C and dissolved. The preset-gel centrifugal disk was stored at 4°C protected from light until use. It was possible to detect 48 genes of two samples simultaneously.

### 2.5. Evaluation of the LCD platform

Total DNA was extracted from 200 μl residual urine samples of 645 patients with suspected UTIs using a genomic DNA extraction kit (Fosun Pharmaceutical Co., Ltd., Shanghai, China), according to the manufacturer’s instructions. Purified nucleic acid was frozen at −80°C until required for verification analysis. ddH_2_O was used as the negative control, and the standard abovementioned strains were used as the positive control. Quality control samples (positive and negative controls) were collected every 10^th^ sample. Briefly, 1 μl Bst 2.0 DNA polymerase (8 U/μL) and 10 μl template were mixed and injected into the injection hole of the sample injection chamber part. Then, the centrifugal disk was inserted into the detection instrument (Figures 2A–C). Finally, the reaction temperature was preset to 63°C for 45 min for isothermal amplification after centrifugation (1,600 rpm for 5 s and 5,000 rpm for 10 s). Data acquisition was performed automatically in real-time *via* the accompanying software. Fluorescence was collected at 30 s intervals. To evaluate the performance of our developed platform in clinical practice for UTI diagnosis, 645 urine samples were simultaneously subjected to LCD and conventional culture.

### 2.6. Evaluation of the specificity and sensitivity of the LCD platform

To evaluate the specificity of the fabricated LCD system among the 24 target genes, various LAMP reaction systems were embedded in the centrifugal disk in advance, as described above. The LAMP reactions were cross-tested among the abovementioned standard strains and negative control, and each experiment was repeated three times.

The sensitivity of the primers in the developed platform for the detection of the 10 pathogens was then determined. Twenty repetitions were conducted of serially diluted templates (organisms diluted in normal urine and then extracted, from 10^8^ to 10^1^ CFU/ml), and each experiment was repeated three times.

### 2.7. DNA amplification and sequencing

Sanger sequencing was performed to assist in the resolution of discordant results between the LCD platform and traditional culture-based methods for identification and AST. DNA was extracted as described previously, and 24 target genes were amplified using the primers outlined in Supplementary Tables S4, S5. Deionized water and the abovementioned bacteria were used as negative and positive controls, respectively. PCR was performed under the following conditions: 94°C for 5 min, 35 cycles of 94°C for 30 s, 58°C for 30 s, 72°C for 1 min, and final annealing at 72°C for 5 min. Subsequently, the PCR products were electrophoresed on 1.2% agarose gel, and the positive products were purified and sequenced by Sangon Biotech Co., Ltd. (Shanghai, China).

### 2.8. Data processing and analysis

To evaluate the results of LCD platform in comparison with other methods as described above. GraphPad Prism 8.0 (GraphPad Prism Software, La Jolla, CA, United States) and SPSS version 20.0 (IBM Corp., Armonk, NY, United States) were used for data processing and analysis. Every target detection result was analyzed using diagnostic test evaluation indices, such as the specificity, sensitivity, positive predictive value (PPV), negative predictive value (NPV), and Cohen’s kappa coefficient (κ) with a 95% CI. The strength of agreement was as follows: κ < 0.40, poor agreement; 0.40 ≤ κ < 0.75, fair to good agreement; and 0.75 ≤ κ < 1.00, excellent agreement. *p*-values ≤0.05 were considered statistically significant. The statistical significance between the developed platform and the traditional method was determined by McNemar’s test, and *p* value <0.05 were considered statistically significant.

## 3. Results

### 3.1. Specificity of the LCD platform for the 24 target genes

LAMP primer optimization is crucial for the platform to be detected accurately and specifically. We next determined the specificity of the 24 developed LAMP assays among Eco, Kpn, Aba, Pae, Sau, Pmi, Ecl, Efa, Efm, *C. albicans*, SHV, TEM, CTX-M-1 group, CTX-M-9 group, KPC, NDM, IMP, OXA-23, OXA-24, OXA-48, DHA, CMY, VanA, and mecA. As shown in Supplementary Figures S1, S2, when the DNA of the corresponding standard strains was added, only the corresponding well had an amplification curve, while the others showed no signal. Additionally, 15 other potential pathogens (namely, *Acinetobacter junii, Acinetobacter johnsonii, Pseudomonas putida, Pseudomonas fluorescens, Klebsielia oxytoca, Klebsiella aerogenes, Serratia marcescens, Staphylococcus epidermidis, Staphylococcus hominis, Staphylococcus saprophyticus, Enterococcus casseliflavus, Enterococcus avium, Candida parapsilosis, Candida glabrata,* and *Candida tropicalis*) were tested and no cross-reactivity was observed, showing that the platform exhibited great specificity for each of the 24 target genes investigated.

### 3.2. Sensitivity of the LCD platform for 10 pathogens

Sensitivity is a critical evaluation index for a detection platform. To determine the sensitivity of the LCD platform for the detection of 10 pathogens (Eco, Kpn, Aba, Pae, Sau, Pmi, Ecl, Efa, Efm, and *C. albicans*), the above 10 ATCC strains were serially diluted with normal urine (1 × 10^8^ CFU/ml to 1 × 10^1^ CFU/ml). As shown in Supplementary Table S6, the Ct of all targets gradually increased with decreasing template concentration. A minimum hit rate of >95% was set as the detection limit. These results indicated that the LCD platform limit of detection was 1 × 10^3^ CFU/ml for the studied pathogens.

### 3.3. Distribution of pathogens detected by the traditional culture approach

In total, 645 samples, from 290 male patients and 355 female patients (age ranging from 21 to 96 years), were collected in our study. A total of 507 strains were isolated from 645 samples with suspected UTIs, including 321 strains of GN bacteria (63.31%), 153 strains of GP bacteria (30.18%), and 33 strains of fungi (6.51%). The GN bacterial population was greater than the GP population, which comprised Eco (34.91%), Kpn (10.85%), Pae (6.51%), Aba (3.94%), Pmi (3.35%), and Ecl (1.78%). The isolated GN bacterial strains predominantly included Efa (17.16%), Efm (7.69%), and Sau (3.55%), while *C. albicans* (5.52%) is the most common fungal pathogen in our study. The other bacteria found at proportions <5% in the samples collected by the culture method were follows: *Klebsiella oxytoca*, *Staphylococcus haemolyticus*, *Staphylococcus epidermidis*, *Streptococcus agalactiae*, *Serratia marcescens*, *Morganella morganii, Candida glabrata*, and *Candida tropicalis.*

### 3.4. Comparison of the LCD platform and culture method for pathogen detection

Currently, midstream urine culture is the gold standard for UTI diagnosis. In this study, 465 samples had a positive pathogen culture outcome, including 423 and 42 samples with mono- and poly-microbial infections, respectively. The LCD platform detected 481 positive cases, 386 of which were single-infection samples and 95 were multiple-infection samples. Table 1 shows the detailed comparative results of the LCD platform and culture for each pathogen studied. The agreement (κ) between these two methods for each pathogen was >0.75, and the developed LCD platform showed good sensitivity and specificity compared to the culture method. According to the result of McNemar’s test, the developed platform and the culture method were statistically different in detecting Eco, Kpn, Aba, Pae, Sau and Pmi (*p* < 0.05). And the positive ratio of developed platform is higher than the culture method.

**Table 1:** Comparison of the coincidence rate of pathogens detection between the LCD platform and culture methods.

Pathogens	Culture	LCD platform		Subtotal	Sensitivity% (95%CI)	Specificity% (95%CI)	McNemar’s test	Kappa test	
		+	−					Kappa	*p*
Eco	+	170	7	177	96.05 (0.917–0.983)	90.38 (0.873–0.928)	*p* < 0.001	0.810	*p* < 0.001
	−	45	423	468					
	Subtotal	215	430	645					
Kpn	+	52	3	55	94.55 (0.839–0.986)	96.27 (0.943–0.976)	*p* < 0.001	0.785	*p* < 0.001
	−	22	568	590					
	Subtotal	74	571	645					
Aba	+	20	0	20	100.00 (0.800–1)	98.24 (0.968–0.991)	*p* = 0.001	0.776	*p* < 0.001
	−	11	614	625					
	Subtotal	31	614	645					
Pae	+	32	1	33	96.97 (0.825–0.998)	98.04 (0.965–0.989)	*p* = 0.003	0.821	*p* < 0.001
	−	12	600	612					
	Subtotal	44	601	645					
Sau	+	17	1	18	94.44 (0.706–0.997)	98.56 (0.972–0.993)	*p* = 0.021	0.765	*p* < 0.001
	−	9	618	627					
	Subtotal	26	619	645					
Pmi	+	16	1	17	94.12 (0.692–0.997)	98.73 (0.974–0.994)	*p* = 0.039	0.773	*p* < 0.001
	−	8	620	628					
	Subtotal	24	621	645					
Ecl	+	9	0	9	100.00 (0.629–1)	99.21 (0.981–0.997)	*p* = 0.063	0.779	*p* < 0.001
	−	5	631	636					
	Subtotal	14	631	645					
Efa	+	77	10	87	88.51 (0.794–0.941)	97.31 (0.955–0.984)	*p* = 0.424	0.838	*p* < 0.001
	−	15	543	558					
	Subtotal	92	553	645					
Efm	+	36	3	39	92.31 (0.780–0.980)	98.18 (0.967–0.990)	*p* = 0.057	0.826	*p* < 0.001
	−	11	595	606					
	Subtotal	47	598	645					
Cal	+	24	4	28	85.71 (0.664–0.953)	98.87 (0.976–0.995)	*p* = 0.549	0.805	*p* < 0.001
	−	7	610	617					
	Subtotal	31	614	645					

Sanger sequencing was conducted on specimens with conflicting results between the LCD platform and culture approach to further verify the diagnostic accuracy of the platform for UTIs. Among the 177 inconsistent samples analyzed by Sanger sequencing, 113 (63.84%) were consistent with our developed platform, including 97 (positive for LCD platform, negative for culture methods) and 16 (positive for culture methods, negative for LCD platform). Table 2 shows that the platform established in this study has excellent specificity to amplify the target pathogens’ DNA in the samples.

**Table 2:** Sanger sequence analysis of 177 inconsistent results between the LCD and culture for pathogens.

Pathogens	Culture/LCD	Sanger sequencing		Total
		+	−	
Eco	+/−	4	3	7
	−/+	30	15	45
Kpn	+/−	1	2	3
	−/+	15	7	22
Aba	−/+	7	4	11
Pae	+/−	1	0	1
	−/+	8	4	12
Sau	+/−	1	0	1
	−/ +	6	3	9
Pmi	+/−	0	1	1
	−/+	4	4	8
Ecl	−/+	4	1	5
Efa	+/−	6	6	12
	−/+	11	4	15
Efm	+/−	1	2	3
	−/+	7	4	11
Cal	+/−	2	2	4
	−/ +	5	2	7

### 3.5. Clinical performance of the LCD platform for antibiotic resistance

Simultaneous detection of 14 target drug-resistant genes from 645 urine samples was conducted using the LCD platform and Sanger sequencing (Table 3). The comparative results showed a high level of agreement between the two methods, with all κ scores >0.8, indicating that the established method had high reliability and accuracy in real clinical samples. Among the drug-resistant genes, six carbapenem drug resistant genes (KPC, NDM, IMP, OXA-23, OXA-24, OXA-48) were detected, among which, KPC was the most abundant (14.11%). Similarly, from the detection rate of the four ESBL resistant genes (SHV, TEM, CTX-M-1 group, CTX-M-9 group), TEM had the highest detection rate in the 645 clinical samples (32.25%).

**Table 3:** Comparison of the developed LCD platform with Sanger sequencing for target ARGs.

ARGs	Sanger sequencing	LCD platform		Subtotal	Sensitivity% (95%CI)	Specificity% (95%CI)	Kappa	*p*
		+	−					
SHV	+	158	7	165	95.76 (0.911–0.981)	96.88 (0.948–0.982)	0.912	*p* < 0.001
	−	15	465	480				
	Subtotal	173	472	645				
TEM	+	199	9	208	95.67 (0.917–0.979)	99.08 (0.975–0.997)	0.954	*p* < 0.001
	−	4	433	437				
	Subtotal	203	442	645				
CTX-M-1 group	+	110	11	121	90.91 (0.840–0.951)	99.24 (0.979–0.998)	0.922	*p* < 0.001
	−	4	520	524				
	Subtotal	114	531	645				
CTX-M-9 group	+	121	7	128	94.53 (0.886–0.976)	98.45 (0.969–0.993)	0.927	*p* < 0.001
	−	8	509	517				
	Subtotal	129	516	645				
KPC	+	89	2	91	97.80 (0.915–0.996)	99.82 (0.988–1)	0.981	*p* < 0.001
	−	1	553	554				
	Subtotal	90	555	645				
NDM	+	16	0	16	100.00 (0.759–1)	99.68 (0.987–0.999)	0.940	*p* < 0.001
	−	2	627	629				
	Subtotal	18	627	645				
IMP	+	4	0	4	100.00 (0.396–1)	100.00 (0.993–1)	1.000	*p* < 0.001
	−	0	641	641				
	Subtotal	4	641	645				
OXA-23	+	11	0	11	100.00 (0.679–1)	99.84 (0.990–1)	0.956	*p* < 0.001
	−	1	633	634				
	Subtotal	12	633	645				
OXA-24	+	6	0	6	100.00 (0.517–1)	100.00 (0.993–1)	1.000	*p* < 0.001
	−	0	639	639				
	Subtotal	6	639	645				
OXA-48	+	3	0	3	100.00 (0.310–1)	100.00 (0.993–1)	1.000	*p* < 0.001
	−	0	642	642				
	Subtotal	3	642	645				
DHA	+	78	3	81	96.30 (0.888–0.990)	99.29 (0.981–0.998)	0.951	*p* < 0.001
	−	4	560	564				
	Subtotal	82	563	645				
CMY	+	18	5	23	78.26 (0.558–0.917)	99.68 (0.987–0.999)	0.832	*p* < 0.001
	−	2	620	622				
	Subtotal	20	625	645				
vanA	+	4	0	4	100.00 (0.396–1)	100.00 (0.993–1)	1.000	*p* < 0.001
	−	0	641	641				
	Subtotal	4	641	645				
mecA	+	10	0	10	100.00 (0.655–1)	100.00 (0.993–1)	1.000	*p* < 0.001
	−	0	635	635				
	Subtotal	10	635	645				

Currently, the phenotypic test (disc diffusion and the MIC method) toward antibiotics of pathogen antibiotics remains the “gold standard” for assessing the AST of strains in clinical practice. We further compared the results of phenotypic and genotypic methods to evaluate the feasibility of the LCD platform to determine drug resistance in an actual sample (Table 4). According to the result of McNemar’s test, the developed platform and the phenotypic test method were statistically different in detecting ESBL and Carbapenem (*p* < 0.05). Of 159 ESBL-positive cases, 148 (93.08%) samples were positive for ESBL genes and predominantly co-expressed multiple resistant genes. However, numerous ESBL genes were also expressed in ESBL negative samples (50.62%). Thus, the kappa statistics of ESBL showed a low congruence between these two methods (kappa value = 0.423). Among the 72 isolates identified as cefoxitin resistant by the phenotypic method, only 33 (45.83%) were identified as carrying plasmid-mediated AmpC genes (DHA and CMY) by the LCD platform. Briefly, the results showed good agreement between the two methods for detecting carbapenem-resistant bacteria, methicillin-resistant bacteria, and vancomycin-resistant bacteria (all kappa value > 0.75), which confirms that the LCD platform could rapidly and effectively detect the most urgent threats of MDR bacteria.

**Table 4:** Results of LCD platform were compared to results using phenotypic tests.

Antibiotic phenotypic tests (numbers)	LCD platform tests (numbers)	PPV% (95%CI)	NPV% (95%CI)	McNemar’s test	Kappa test	
					Kappa	*p*
ESBLs+ (159)	SHV + TEM + CTX-M (51)	64.35 (0.577–0.704)	87.91 (0.790–0.935)	*p* < 0.001	0.423	*p* < 0.001
	SHV + CTX-M (14)					
	TEM + CTX-M (43)					
	SHV + TEM (5)					
	SHV (4)					
	TEM (6)					
	CTX-M (25)					
	Negative (11)					
ESBLs- (162)	SHV + TEM + CTX-M (15)					
	SHV + CTX-M (17)					
	TEM + CTX-M (4)					
	SHV + TEM (17)					
	SHV (10)					
	TEM (14)					
	CTX-M (5)					
	Negative (80)					
Carbapenem resistant (84)	KPC + NDM (2)	76.19 (0.667–0.837)	98.15 (0.950–0.994)	*p* < 0.001	0.784	*p* < 0.001
	OXA-23 + OXA-24 (5)					
	KPC (54)					
	NDM (8)					
	IMP (3)					
	OXA-23 (5)					
	OXA-48 (3)					
	Negative (4)					
Carbapenem sensitive (237)	KPC (21)					
	NDM (2)					
	OXA-23 (2)					
	Negative (212)					
Cefoxitin-resistant GN (72)	DHA + CMY (9)	56.90 (0.433–0.696)	80.69 (0.744–0.858)	*p* = 0.103	0.346	*p* < 0.001
	DHA (20)					
	CMY (4)					
	Negative (39)					
Cefoxitin-sensitive GN (188)	DHA + CMY (2)					
	DHA (22)					
	CMY (1)					
	Negative (163)					
Vancomycin resistant (4)	vanA (4)	100.00 (0.396–1)	100.00 (0.969–1)	*p* = 1.000	1.000	*p* < 0.001
	Negative (0)					
Vancomycin sensitive (149)	vanA (0)					
	Negative (149)					
Cefoxitin-resistant GP (9)	mecA (9)	90.00 (0.541–0.995)	100.00 (0.717–1)	*p* = 1.000	0.911	*p* < 0.001
	Negative (0)					
Cefoxitin-sensitive GP (14)	mecA (1)					
	Negative (13)					

PPV, positive predictive value; NPV, negative predictive value; GN, Gram-negative; GP, Gram-positive.

## 4. Discussion

Currently, laboratories assayed each bacteria and AST using their routine clinical assays, however, these methods have several drawbacks, including low sensitivity (Paudel et al., 2020) and a long reaction time. Therefore, there remains an urgent need for improved diagnostics, including pathogen identification and antimicrobial-resistance profiling (Mach et al., 2011). As investigation into the mechanism of antibiotic resistance deepens, the roles of molecular biology techniques in infectious diseases have gained increasing attention.

LAMP is a technology that combines a straightforward, rapid (within 45 min), affordable, highly sensitive, and specific detection method. We previously optimized the LAMP reaction conditions and validated the detection ability of LAMP for respiratory tract infection (Chen et al., 2021; Si et al., 2021). Given the demand for clinical diagnosis, we established a rapid, simple, and sensitive detection platform combining LAMP and the centrifugal disk, which is applicable to POCT to facilitate the accurate detection of UTIs. With no requirement for prior isolation of monoclonal strains, we describe the sensitivity, capability, and accuracy of the developed LCD platform to perform rapid pathogen and antibiotic-resistance testing in clinical urine samples from patients with suspected UTIs.

A total of 507 strains were detected in 645 clinical samples, among which, GN strains were dominant, especially Eco (34.91%). Efa (17.16%), and Efm (7.69%) were the predominant species of GP strains. These Enterococcus and Eco species, which comprise the normal intestinal flora, are opportunistic human pathogens, indicating that enteric bacteria remain the most frequent cause of UTIs. The culture method requires a viable pathogen and often only the dominant colony can be cultured. Therefore, the developed LCD platform was also superior in terms of sensitivity in detecting samples with multiple infections. The κ > 0.75 of all detected pathogens in UTIs indicated a good agreement between the LCD platform and the traditional culture method, which was higher than our previous detection in LRTI (Si et al., 2021). This may be attributable to the respiratory tract environment, which differs from the urinary tract milieu in that the microbial colonization is much more limited in the urinary tract. Li et al. suggests the culture method that provides false-negative results as the primary cause of the difference between the two approaches for detecting infections (Li et al., 2017). All discordant results between the LCD platform and culture method were substantially illustrated by Sanger sequencing, and the agreement of the inconsistent samples between the LCD platform and Sanger sequencing was significantly higher than that between the culture method and Sanger sequencing. Indeed, the limitation of the LCD platform is that it can only detect specific targeted pathogens and cannot be used for all pathogens. Nevertheless, the results of the LCD platform revealed high sensitivity and specificity for pathogens, which suggests that the assay is appropriate for rapid and effective detection of the microorganisms in low concentration.

Obtaining antibiotic resistance results in as short a time as possible is crucial for patients. We developed a portable device to detect the 14 main resistant genotypes of the concern MDR bacteria spreading in China simultaneously. The analytical sensitivity and specificity of the device were more than 0.8, taking Sanger sequencing as the gold-standard method for comparison, which indicates that the primers used in this assay are suitable for the rapid detection of target ARGs. Due to the complex resistance mechanisms, the presence or absence of a resistance gene is not always sufficient to accurately predict the resistance phenotype. The differences results in antibiotic-resistant between culture and molecular methods are well recognized now (Keane et al., 2013), we sought to further compare them in our research.

Several studies have described molecular biology techniques, including high-throughput next-generation sequencing, PCR, and LAMP, for detecting carbapenemase activity (Ngamsaad and Khompurngson, 2012). Production of carbapenemases is one of the main mechanisms underlying the resistance of carbapenem-resistant strains in China, among which, KPC and NDM are the major carbapenemases of CRE (Zhang et al., 2017). OXA-type carbapenemase genes are considered the primary underlying cause of CRAB and CRPA, especially OXA-23 (Aruhomukama et al., 2019; Huang et al., 2019). Based on the actual situation in China, we detected the six main carbapenemase genes in parallel. When comparing the phenotypic test results with the LCD test results, we found an excellent strength of agreement between the two tests (kappa value = 0.784). Additionally, there was good agreement between the two test outcomes in ESBL-related resistant genes. ESBL genes (SHV, TEM, and CTX-M) were detected in 93.08% of ESBL-positive samples, while a high proportion (50.62%) was detected by the LCD platform in culture-negative specimens. Hence, the high NPV (87.91%) of this assay indicated that it could be used as an effective method to detect ESBL non-producers. However, the LCD platform showed poor agreement for determining AmpC. In our research, AmpC phenotypes were estimated based on antimicrobial susceptibility to cefoxitin, which may explain why the LCD platform had poor performance in AmpC. DHA and CMY are the most common type of plasmid-mediated AmpCβ-lactamase in China. This study revealed that the predominant mechanism of acquiring AmpC-type resistance was chromosomal-mediated rather than plasmid-mediated AmpC genes. Further studies should be conducted on chromosomal-mediated AmpC to improve the detection ability of AmpC producers.

A limited number of resistance mechanisms are available for GP bacteria. Our results suggest that the LCD platform is an effective approach for identifying MRSA and VRE (kappa value >0.9). It is also important to note that the number of positives in VRE may restrict the performance of the LCD platform. Nevertheless, molecular analysis is an effective analytical method for the detection of urgent concern MDR in GP bacteria.

Multiplex molecular assays targeting numerous pathogens directly from clinical specimens have led to a paradigm shift in the diagnosis of infectious diseases (Ramanan et al., 2018). Systems from Luminex (Gadsby et al., 2010; Butt et al., 2014; Lee et al., 2017), GenMark (Pierce and Hodinka, 2012), and bioMérieux (Poritz et al., 2011) are among the multiplex nucleic acid-based assays for infectious agents that are commercially accessible and FDA approved. All such systems combine the nucleic acid purification and multiplex PCR amplification. The platform developed in this study performs under isothermal conditions, which can simultaneously provide potential pathogens and ARGs. More data need to be collected to further illustrate “false positive results” that could come to the limit of detection of the reference culture method from “false positive result” that could be linked to dead bacteria or free DNA.

UTIs are some of the most common infectious diseases that affect people of all ages. Many pathogens are known to cause UTIs in patients with similar clinical symptoms. Hence, relying on clinical symptoms and routine urine examination findings to diagnose UTIs may lead to misdiagnosis or unnecessary treatment. Moreover, the irrational use of antibiotics has led to a steady increase in MDR bacteria. Our data suggest that the developed platform provides a valuable diagnostic adjunct for UTIs. The LAMP method does not require cyclic temperature changes and can be conducted at a constant temperature using simple instruments, which drastically reduces equipment costs. We also designed and manufactured a centrifugal disk system (length × width × height: 170 mm × 170 mm × 170 mm) that is small, easy to move, costs much less than a traditional fluorescence amplification instrument, and can be operated by trained laboratory personnel. Importantly, the platform not only has high accuracy but also meets the need of rapid diagnosis, which can be completed within 1.5 h of specimen collection. Hence, once the protocol has been established, it is a fast and easy method. These tests spent an average of US$12 for every sample. Alternatively, costs can be further reduced, if their storage and delivery can be optimized, which is an important technical challenge to solve.

Nevertheless, the limitation of this study is that only qualitative assessment could be performed, and the proposed LCD device does not exactly allow the direct processing of clinical samples, as it processes extracted DNA. Thus, the described method is not yet adapted to POCT as it requires a separate step of nucleic acids extraction. The above would be a fruitful area for further work. Additionally, as we did not assess clinical data, we do not know whether any of these may have been contaminants in the urine sample. Clearly, additional clinical trials should be conducted to guide the clinical use of medication and to further evaluate the feasibility of the method based on the recovery of the patient after administration.

## 5. Conclusion

We have developed a platform that can be used to detect the majority of pathogens and the urgent concern MDR from patient samples with suspected UTIs. This platform can be operated in a closed system without opening lids, which significantly reduces the need for multi-step sample manipulation and prevents potential cross-contamination. Beyond this, the platform showed high diagnostic efficacy among clinical samples. Our findings offer a powerful tool for evidence-based UTI diagnosis, which has important implications for the rational use of antibiotics.

With deepening research on resistance mechanisms and the accumulation of clinical data, the relationship between bacterial genotype and drug-resistance becomes more explicit. We believe that the molecular-based identification of causative pathogens and their resistance will become a major trend in diagnosis.

### 5.1. Importance

- The whole process, from sample analysis to result read out can be achieved in 1.5 h.
- The centrifugal disk system can detect 10 pathogens and 24 resistant genes simultaneously.
- A total of 645 urine samples were analyzed to evaluate the diagnosis ability of the developed platform.

## Data availability statement

The data presented in the study are deposited in the GenBank repository, accession number OQ675710-OQ675822, further inquiries can be directed to the corresponding author.

## Ethics statement

The protocol was approved by the “Ethics commission of the Shanghai East Hospital” under the protocol no. 2019-021. Written informed consent for participation was not required for this study in accordance with the national legislation and the institutional requirements.

## Author contributions

NC: conceptualization, methodology, data curation, validation, and writing the original draft. GL: data curation, validation, and conceptualization. YS: resources, investigation, and conceptualization. YY: data curation and validation. TZ and DC: instrument operation. WZ, LP, GQ, and YL: resources and investigation. MZ: supervision. GS: conceptualization and funding acquisition. LF: conceptualization, funding acquisition, and writing review and editing. All authors contributed to the article and approved the submitted version.

## Funding

This work was supported by Pudong New Area Construction of Key Disciplines of the Health and Family Planning Commission (grant number: PW2019E-2); Shanghai Science and Technology Commission Science and Technology Innovation Action Plan (grant number: 21Y11900800); the National Natural Science Foundation of China (grant number: 81971535); Shanghai Municipal Health Commission (grant number: YYXX202101); Public Health Highland Subject of Pudong Health Commission of Shanghai (grant number: PWYggy2021-01); the Science and Technology Commission of Shanghai Municipality (grant number: 23YF1434600).

## Conflict of interest

DC is employed by Fosun Diagnostics Co., Ltd.

The remaining authors declare that the research was conducted in the absence of any commercial or financial relationships that could be construed as a potential conflict of interest.

## Publisher’s note

All claims expressed in this article are solely those of the authors and do not necessarily represent those of their affiliated organizations, or those of the publisher, the editors and the reviewers. Any product that may be evaluated in this article, or claim that may be made by its manufacturer, is not guaranteed or endorsed by the publisher.

## Footnotes

^1^ http://primerexplorer.jp/lampv5e/index.html
