# Supplementary material for: Development and evaluation of a centrifugal disk system for the rapid detection of multiple pathogens and their antibiotic resistance genes in urinary tract infection
Source: Front Microbiol. 2023 May 2;14:1157403. doi: 10.3389/fmicb.2023.1157403 (PMC10187633; doi:10.3389/fmicb.2023.1157403)
Supplement: Supplementary file 1 [file Data_Sheet_1.PDF]

Supplemental Material

Table S1

Bacterial strains producing the following representative enzyme were used as controls.

Strain No.	Strain Species	Antibiotic Resistant Genes	Enzyme family (Ambler classification)
1	Kpn	SHV-1	Penicillinase (A)
2	Eco	SHV-28	Penicillinase (A)
3	Eco	TEM-1	Penicillinase (A)
4	Kpn	CTX-M-3	Extended spectrum β-lactamases (A)
5	Kpn	CTX-M-15	Extended spectrum β-lactamases (A)
6	Eco	CTX-M-55	Extended spectrum β-lactamases (A)
7	Eco	CTX-M-64	Extended spectrum β-lactamases (A)
8	Eco	CTX-M-14	Extended spectrum β-lactamases (A)
9	Kpn	KPC-2	Carbapenemase (A)
10	Kpn	NDM-1	Carbapenemase (B)
11	Serratia marcescens	IMP-8	Carbapenemase (B)
12	Aba	OXA-23	Carbapenemase (D)
13	Aba	OXA-24	Carbapenemase (D)
14	Kpn	OXA-48	Carbapenemase (D)
15	Pmi	DHA-1	Cephalosporinase (C)
16	Eco	CMY-2	Cephalosporinase (C)
17	Efa	VanA	-
18	Efm	VanB	-
19	Sau	mecA	-

Table S2

LAMP primer sets used for target pathogens

Target pathogens	Target gene	Primer	Sequence (5'→3')
Eco	malk	F3	TGCCAGTTTCAGGCCAAAT
		B3	TCTGGCTGCGGTAAATCGA
		FIP	CAGCAGAACGTGGCGTTGGTATTTTGCTTTTTACTGACAGGTGGGGA
		BIP	TCTCACCGATGAACAGGTCGCTTTCTTTTTTTACTGCGCATGATTGCC
		LF	GTTTCAGTCCTACGCGCTCT
		LB	CTGGTGATCGTCTCAAGCCC
Kpn	PhoE	F3	CCGACATTGTCACTGAGT
		B3	CCTGAATACCGGAGGTGAT
		FIP	GCTTTGATGTTCATTTGCGTTGAGACATTTTAATATGCTGCAAATGTGG
		BIP	GCTTTGATGTTCATTTGCGTTGAGACATTTTAATATGCTGCAAATGTGG
		LF	TTTGCGAAGTACCATGCC
Aba	ITS	F3	GGATCACCTCCTTAACGAA
		B3	CAGCTTACATCATCAATCATGT
		FIP	GCTACAGACCCTCAGATACATCTAGATTTTTTGACGATTGGTAAGAATCC
		BIP	CAGTTGGTTAGAGCACACGCTCAACCTTTTAGTCAAAGTCATGG
		LB	TGATAAGCGTGGGGTCACA
Pae	Exotoxin A	F3	GCGCGATGCCACCTTCT
		B3	TGCAGCGTTGCTGGGC
		FIP	CCATGACCACGCTGACCCCGTTTTCATTTTCGAGAGCAACGAGATGC
		BIP	CGGGAAAAGCGCTGGAGCGAATTTTTTTTGAGGTAGTTGTAGACCCCGT
		LB	GCAAGGTGTTGTGCCTGCTC
Sau	nuc	F3	CGCTACTAGTTGCTTAGTGTT
		B3	TGTCATTGTTTGACCTTTGT
		FIP	CGCCGTTACCTGTTTGTGATACTTTAGTTTTTGTAGCTTCAAGTC
		BIP	AGATCCAACAGTATACAGTGCAACTCGTTTTTATCACCATCAATCGCTT
		LF	AAAAATTACATAAAGAACCTGCG
Pmi	ureC	F3	GTTACCCCCGGTATTTGGAA
		B3	CATCGGCGACATTAAGGCA
		FIP	GGCTGACTGACACAACCTTTGCTTTTTGTTAGAGGCGGTGGATGA
		BIP	TCCGCGAACAAATAACAGCGGGTTTTAATTGCCATTGGCGTTGC
Ecl	ompX	F3	AACGACTGGGCAAGCATC
		B3	TAACCTACGCCCGCGATC
		FIP	GCGGTTCAGACCCTGGTTTTCGTTTTTACGGTGTTGTAGGTGTTGG
		BIP	GCGGGTCTGCAGTTCAACCCTTTTTCAACGTTGCGGATACGG
		LB	AATCGAAGACGTTGCTCTGGA
Efa	ddl	F3	GCACGTGAAATTGAAGTAGC
		B3	ACTTCCATCTAACATAATATACGC
		FIP	AAGCGACATCTTTCACCACTTCTTTTCATTTTAGGAAATGAAGATGTCCG
		BIP	ACATCAATAACACGATTGAAATGCATTTTTTCTTGCGCTTGATGAGC
		LB	AGCGCATGTTCCAGAAGAAG
Efm	ddl	F3	ATGGGTCAAAGGTCCATT
		B3	TCAATGTCTCTAAGAAGCCTT
		FIP	CTGTCTGTCCACTTGGGTCCTTTTACAGAAAAACCTGCCAGC
		BIP	AAGTGATCAATCCGGGCGAAATTTTTTCCATCTTCCCCGTTTG
		LB	GAGCCATCGTTTTTCCAGTTTTAC
C.albicans	Hwp1	F3	TCCATTGACTGAAACTACTCC
		B3	TCAGTCAATGGACAGAATGT
		FIP	AGTTGATTGTTCAGTAGAAGTGGATTTTTGTTTCTACTGCTCCAGCC
		BIP	GTTCTGAAAGCTCTTGTACCGAATTTTAGTGTAGACAGTTTCCTCAG
		LB	TCTGAAGTTACTACTGGTGTTGTTG

Table S3

LAMP primer sets used for target antibiotic resistant genes

Target gene	Primer	Sequence (5'→3')
SHV	F3	CACTATCGCCAGCAGGATC
	B3	GACGTTGTCGCCGATCTG
	FIP	TTCGCCGACCGTCATGCCGTTTTTGGTGGACTACTCGCCGG
	BIP	TACCATGAGCGATAACAGCGCCTTTTCGCAAAAAGGCAGTCAATCC
	LB	CCAATCTGCTGCTGGCCACC
TEM	F3	AGGACCGAAGGAGCTAACC
	B3	TGGTCCTGCAACTTTATCCG
	FIP	GGCTTCATTCAGCTCCGGTTCCTTTTTTGCACAACATGGGGGAT
	BIP	CACCACGATGCCTGCAGCAATTTTTTGTTGCCGGGAAGCTAGA
CTX-M-1 group	F3	TTCCGTCTCGACCGTACC
	B3	AGGAAGCAGGCAGTCCAG
	FIP	AGTTTGCGCCATTGCCCGAGTTTTTAAACACCGCCATTCCGG
	BIP	GGTAAAGCATTGGGCGACAGCTTTTCTGAATGCTCGCTGCACC
	LF	TGGTATCACGCGGATCGC
	LB	GGCGCAGCTGGTGACAT
CTX-M-9 group	F3	GGAGAAAAGCAGCGGAGG
	B3	GGCTTGATCTCGACAGGC
	FIP	GGAAAGCGTTCATCACCGCGATTTTTCGTCGCGCTCATCGATAC
	BIP	ACCAGTAAAGTTATGGCGGCCGTTTTAGCAGCTGCTTTTGCGTT
	LB	CGGCGGTGCTTAAGCAGAG
KPC-2	F3	GGCTCAGGCGCAACTG
	B3	GGGTGACCACGGAACCA
	FIP	CGGCAGCAAGAAAGCCCTTGAATTTTTAAGTTACCGCGCTGAGGA
	BIP	TGTGCTGGCTCGCAGCCATTTTGCGCATTTTTGCCGTAACGG
NDM	F3	TCGACGGCACCGACATC
	B3	TTGCGGCGCGGCTATC
	FIP	GGCATCACCGAGATTGCCGAGCTTTTCTTTTGGTGGCTGCCTGATC
	BIP	CACTGAGCACTACGCCGCGTTTTTCGGAATGGCTCATCACGAT
	LF	ACTTGGCCTTGCTGTCCTT
	LB	CGTTTGGTGCGGCGTTC
IMP	F3	GACACTCCATTTACGGCTA
	B3	TTTTAGCTTGAACCTTACCG
	FIP	ATGAAAATGAGAGGAAATACTGCCTTTTTGAAAAGTTAGTCACTTGGTTTGT
	BIP	GGCGGAATAGAGTGGCTTAATTTTTTTCATTAGTTAATTCAGACGCATAC
	LF	TGTCAATTAGATAAGCTTCA
	LB	GTGGCTATAAAATAAAAGGC
OXA-23	F3	TGTTCAAGGACATAATCAGGT
	B3	TTTTCTCGCCCTTCCATT
	FIP	GCGGCTTAGAGCATTACCATATAGATTTTTACTTTGATGAAAAAAACACCTC
	BIP	GCAAATACAGAATATGTGCCAGCTTTTTAATATCCGTTTTCTGGTTCTC
	LF	TGTTTGAATAACCAGCACACCT
OXA-24	F3	AAGCTCAAACACAGGGTG
	B3	AGTCATATCTTTCTCCCACAT
	FIP	TGTTGATGCAGGGACATATTCTTTATTTTAGAGGGTAAAAATCTTAGCACC
	BIP	ATGCTTTAATCGGGCTAGAAAATCATTTTGGATAAGTTCTTTTTTTACCATCC
	LF	CTCGTGCAAGAGCATTACCATA
OXA-48	F3	AATAGCTTGATCGCCCTC
	B3	CCATAATCGAAAGCATGTAGC
	FIP	GATTCCAAGTGGCGATATCGCTTTTGGCGTGGTTAAGGATGAAC

	BIP	TAATCACCGCGATGAAATATTCAGTTTTTCTTGCTCATACGTGCCTC
	LF	TGTCCATCCCACTTAAAGACTTG
	LB	TGTGCCTGTTTATCAAGAATTTGCC
DHA	F3	ACTATAAAACCGCCGCGATT
	B3	GCGCCGAAACCAGTTGTT
	FIP	GACCTCGTTGGTCACACCGTTATTTTGGGCTGGGAAATGTATGACT
	BIP	ATTGCAGCCGCATCCGGTAATTTTTTTTATGCACCCAGGAAGCA
	LB	CAGACAACCAGGTTCAGCCG
CMY	F3	GCGATCCGGTCACGAAAT
	B3	CATTCCTGAGGGTTTCACCG
	FIP	GTAGGCCGCCTGCCGTATAGTTTTGCAAACAGTGGCAGGGTA
	BIP	ATCAAAACTGGCAGCCGCAATGTTTTCCAATGCTGGAGTTAGCGTA
VanA	F3	TAGCGCGGACGAATTGGA
	B3	CGGCTCGACTTCCTGATGA
	FIP	GCCCGAAACAGCCTGCTCAATTTTTTACGCAATTGAATCGGCAAGA
	BIP	TGTGAGGTCGGTTGTGCGGTATTTTTGCAGCCTGATTTGGTCC
	LB	TTGGGAAACAGTGCCGCGTTA
mecA	F3	GGATGAATATTTAAGTGATTTCGCA
	B3	GAGCTTTTTATCGTAAAGTTTTTCG
	FIP	ACCTAATAGATGTGAAGTCGCTTTTTTTTCTTACAACTAATGAAACAGAAAGTC
	BIP	TATGTTGGTCCCATTAACTCTGAAGTTTTCCCTTTTTACCAATAACTGCA
	LF	CCTAGAGGATAGTTAC

Table S4

Primer sets used in the Sanger sequencing for pathogens

Target pathogens	Target genes	Primer	Sequence (5'→3')	Length
Eco	malk	Forward	GAAACACCATACCAACGCCACG	389bp
		Reverse	TCACAGAAGCCGCGTTCTCA	
Kpn	PhoE	Forward	TGCCCAGACCGATAACTTTA	142bp
		Reverse	CTGTTTCTTCGCTTCACGG	
Aba	ITS	Forward	CGTCGGTCGGATCCGTGTAT	585bp
		Reverse	AAGTAAAGTGGCAGGCGCTT	
Pae	Exotoxin A	Forward	TACCTTGCTGTTTTGAC	137bp
		Reverse	ATCCAACTTGCTGAACCAC	
Sau	nuc	Forward	TGATACACCTGAAACAAAGCATCC	202bp
		Reverse	TTGACGAACTAAAGCTTCGTTTACC	
Pmi	ureC	Forward	TGTCCACAACAAGCCCA	309bp
		Reverse	TACATCCATTTCATCGG	
Ecl	ompX	Forward	GCAGGATAACAACCCAC	289bp
		Reverse	GGAGAAGTCCAGAGCAA	
Efa	ddl	Forward	TGAACGACCACAAAATA	301bp
		Reverse	TAAAACTGGCACGAATG	
Efm	ddl	Forward	GTGTCCATTCTTTCCGC	283bp
		Reverse	AGCCTTTGATCGTTCCA	
C.albicans	Hwp1	Forward	AGGGAGAGTTTTGGTAGG	712bp
		Reverse	CTTGTGGCTGTTGTGGAT	

Table S5

Primer sets used in the Sanger sequencing for antibiotic resistant genes

Target gene	Primer	Sequence (5'→3')	Length
SHV	Forward	CTCCCTGTTAGCCACCCT	477bp
	Reverse	GCCTCATTCAGTTCCGTTT	
TEM	Forward	ATGAGTATTCAACATTTCCGTG	861bp
	Reverse	TTACCAATGCTTAATCAGTGAG	
CTX-M-1 group	Forward	CGTCACGCTGTTGTTAGGAA	780bp
	Reverse	ACGGCTTTCTGCCTTAGGTT	
CTX-M-9 group	Forward	TACGCAGGTGCTTTATCG	481bp
	Reverse	CGTATTGCCTTTGAGCC	
KPC	Forward	CCGACGCCTTGCCAATTGCAGA	656bp
	Reverse	CCGCCGCCAATTTGTTGCT	
NDM	Forward	ATGTCTGGCAGCACACTTCC	650bp
	Reverse	AGGTTATCAGCGCAGCTTGT	
IMP	Forward	TGATTGACACTCCATTTAC	445bp
	Reverse	ACAACCAGTTTTGCTTTAC	
OXA-23	Forward	CTATGTGGTTGCTTCTCTTT	614bp
	Reverse	TGTAGCCATTACTCTCTTCT	
OXA-24	Forward	GCACGAGCAAATAAAGAAT	541bp
	Reverse	CTAAGTTGAGCGAAAAGGG	
OXA-48	Forward	TTG GTGGCATCGATTATCGG	744bp
	Reverse	GAGCACTTCTTTTGTGATGGC	
DHA	Forward	AACTTTCACAGGTGTGCTGGGT	405bp
	Reverse	CCGTACGCATACTGGCTTTGC	
CMY	Forward	TGGCCAGAACTGACAGGCAAA	462bp
	Reverse	TTTCTCCTGAACGTGGCTGGC	
VanA	Forward	AGGAGCATGACGTATCGG	630bp
	Reverse	CGGCACTGTTTCCCAAT	
mecA	Forward	CGTTCCACTTAAAACCGTTAA	229bp
	Reverse	GTAAAGTTTTTCGAGTCCCTT	

Table S6

Confirmation of limit of detection of targeted pathogens processed in LCD platform

Sample tested in LCD	DNA concentration (CFU/ml)	No. Of replicates with positive amplification (%)	Mean Ct value*	SD*
	1x10^8	20/20 (100%)	9.00	0.78
	1x10^7	20/20 (100%)	14.51	0.91
	1x10^6	20/20 (100%)	23.49	0.80
Eco (ATCC 35218)	1x10^5	20/20 (100%)	28.58	1.27
diluted in normal urine	1x10^4	20/20 (100%)	32.73	1.28
	1x10^3	14/20 (70%)	37.56	0.92
	1x10^2	2/20 (10%)	40.40	0.23
	1x10^1	0/20 (0%)	ND	ND
	1x10^8	20/20 (100%)	14.26	0.85
	1x10^7	20/20 (100%)	20.00	1.00
	1x10^6	20/20 (100%)	24.23	1.42
Kpn (ATCC 13883)	1x10^5	20/20 (100%)	29.29	0.80
diluted in normal urine	1x10^4	20/20 (100%)	33.24	1.02
	1x10^3	16/20 (80%)	39.04	0.49
	1x10^2	0/20 (0%)	ND	ND
	1x10^1	0/20 (0%)	ND	ND
	1x10^8	20/20 (100%)	14.51	0.86
	1x10^7	20/20 (100%)	20.43	1.17
	1x10^6	20/20 (100%)	24.16	1.46
Aba (ATCC 19606)	1x10^5	20/20 (100%)	29.47	0.87
diluted in normal urine	1x10^4	20/20 (100%)	32.55	1.16
	1x10^3	17/20 (85%)	38.88	0.61
	1x10^2	1/20 (5%)	41.48	ND
	1x10^1	0/20 (0%)	ND	ND
	1x10^8	20/20 (100%)	15.03	1.32
	1x10^7	20/20 (100%)	20.63	0.85
	1x10^6	20/20 (100%)	25.38	0.81
Pae (ATCC 27853)	1x10^5	20/20 (100%)	28.38	1.01
diluted in normal urine	1x10^4	20/20 (100%)	32.75	1.18
	1x10^3	18/20 (90%)	39.01	0.61
	1x10^2	4/20 (20%)	43.83	1.29
	1x10^1	0/20 (0%)	ND	ND
	1x10^8	20/20 (100%)	9.38	0.87
	1x10^7	20/20 (100%)	18.69	0.91
	1x10^6	20/20 (100%)	22.97	0.84
Sau (ATCC 25923)	1x10^5	20/20 (100%)	28.92	1.35
diluted in normal urine	1x10^4	20/20 (100%)	33.11	1.04
	1x10^3	13/20 (65%)	38.01	0.70
	1x10^2	1/20 (5%)	42.04	ND
	1x10^1	0/20 (0%)	ND	ND
Pmi (ATCC 12453)	1x10^8	20/20 (100%)	12.34	0.89
diluted in normal urine	1x10^7	20/20 (100%)	14.53	0.81

	$1x10^6$	20/20 (100%)	18.68	0.85
	$1x10^5$	20/20 (100%)	28.39	0.90
	$1x10^4$	20/20 (100%)	32.76	0.94
	$1x10^3$	18/20 (90%)	33.48	0.97
	$1x10^2$	8/20 (40%)	43.26	0.91
	$1x10^1$	0/20 (0%)	ND	ND
Ecl (ATCC 700323) diluted in normal urine	$1x10^8$	20/20 (100%)	13.00	0.87
	$1x10^7$	20/20 (100%)	17.62	0.80
	$1x10^6$	20/20 (100%)	23.66	0.91
	$1x10^5$	20/20 (100%)	29.37	1.30
	$1x10^4$	20/20 (100%)	32.34	0.86
	$1x10^3$	19/20 (95%)	32.56	0.93
	$1x10^2$	7/20 (35%)	38.87	1.39
	$1x10^1$	0/20 (0%)	ND	ND
Efa (ATCC 29212) diluted in normal urine	$1x10^8$	20/20 (100%)	14.73	0.72
	$1x10^7$	20/20 (100%)	18.90	0.66
	$1x10^6$	20/20 (100%)	23.60	1.11
	$1x10^5$	20/20 (100%)	29.00	1.19
	$1x10^4$	20/20 (100%)	33.11	1.11
	$1x10^3$	15/20 (75%)	37.53	0.93
	$1x10^2$	3/20 (15%)	42.84	0.10
	$1x10^1$	0/20 (0%)	ND	ND
Efm (ATCC 35667) diluted in normal urine	$1x10^8$	20/20 (100%)	13.57	0.71
	$1x10^7$	20/20 (100%)	17.57	0.85
	$1x10^6$	20/20 (100%)	23.72	0.83
	$1x10^5$	20/20 (100%)	28.82	1.24
	$1x10^4$	20/20 (100%)	32.66	0.79
	$1x10^3$	14/20 (70%)	40.66	0.86
	$1x10^2$	0/20 (0%)	ND	ND
	$1x10^1$	0/20 (0%)	ND	ND
C.albicans (ATCC 49766) diluted in normal urine	$1x10^8$	20/20 (100%)	13.50	0.65
	$1x10^7$	20/20 (100%)	17.49	0.65
	$1x10^6$	20/20 (100%)	23.44	0.96
	$1x10^5$	20/20 (100%)	29.07	1.18
	$1x10^4$	20/20 (100%)	32.40	0.83
	$1x10^3$	13/20 (65%)	40.56	0.92
	$1x10^2$	0/20 (0%)	ND	ND
	$1x10^1$	0/20 (0%)	ND	ND

*Values were calculated using GraphPad Prism; ND means not detected and no pathogens' amplification observed.

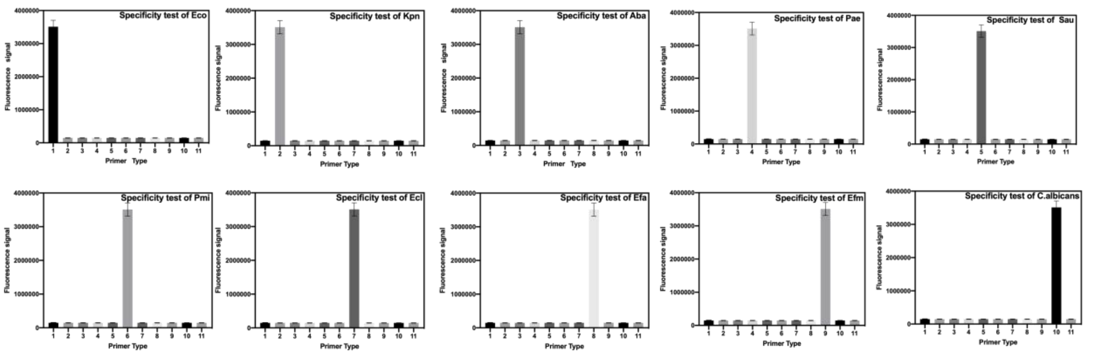

Figure S1

Results of the specific evaluation of the different pathogens samples by different primers. Abscissa: 1, Eco primer; 2, Kpn primer; 3, Aba primer; 4, Pae primer; 5, Sau primer; 6, Pmi primer; 7, Ecl primer; 8, Efa primer; 9, Efm primer; 10, C.albicans primer; 11, negative control.

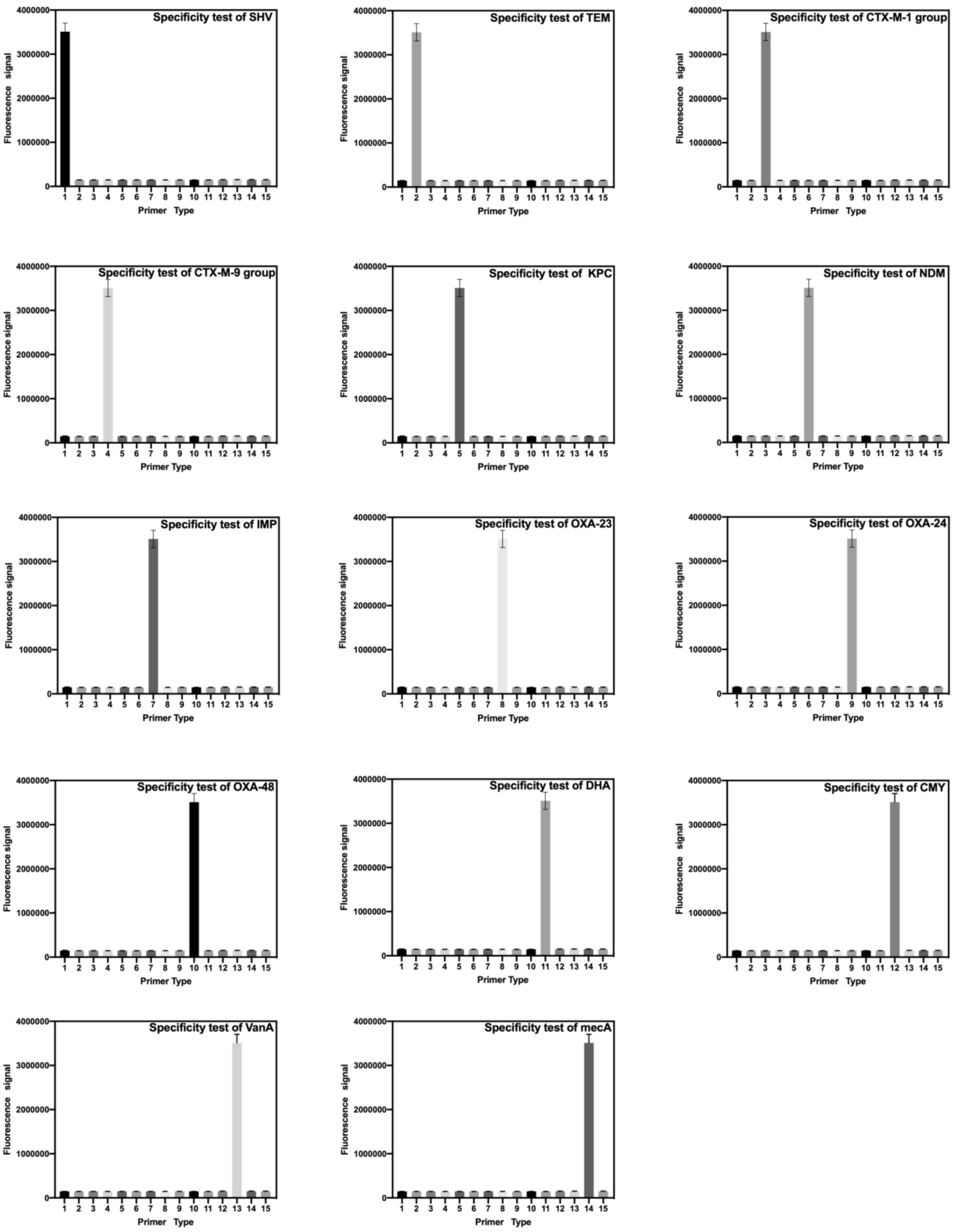

Figure S2

Results of the specific evaluation of the different resistant genes samples by different primers. Abscissa: 1, SHV primer; 2, TEM primer; 3, CTX-M-1 group primer; 4, CTX-M-9 group primer; 5, KPC primer; 6, NDM primer; 7, IMP primer; 8, OXA-23 primer; 9, OXA-24 primer; 10, OXA-48 primer; 11, DHA primer; 12, CMY primer; 13, VanA primer; 14, mecA primer; 15, negative control.
